# Fish oil administration in older adults: is there potential for adverse events? A systematic review of the literature

**DOI:** 10.1186/1471-2318-13-41

**Published:** 2013-05-01

**Authors:** Anthony M Villani, Maria Crotty, Leslie G Cleland, Michael J James, Robert J Fraser, Lynne Cobiac, Michelle D Miller

**Affiliations:** 1 Department of Nutrition and Dietetics, School of Medicine, Flinders University, Adelaide, South Australia; 2 Department of Rehabilitation and Aged Care, School of Medicine, Flinders University, Adelaide, South Australia; 3 Rheumatology Unit, Royal Adelaide Hospital, Adelaide, South Australia; 4 Flinders Clinical Effectiveness, School of Medicine, Flinders University, Adelaide, South Australia

**Keywords:** Fish oil, Eicosapentaenoic acid, Docosahexaenoic acid, Adverse events, Older adults

## Abstract

**Background:**

Omega-3 (n-3) fatty acid supplementation is becoming increasingly popular. However given its antithrombotic properties the potential for severe adverse events (SAE) such as bleeding has safety implications, particularly in an older adult population. A systematic review of randomized control trials (RCT) was conducted to explore the potential for SAE and non-severe adverse events (non-SAE) associated with n-3 supplementation in older adults.

**Methods:**

A comprehensive search strategy using Medline and a variety of other electronic sources was conducted. Studies investigating the oral administration of n-3 fish oil containing eicosapentaenoic acid (EPA), docosahexaenoic acid (DHA) or both against a placebo were sourced. The primary outcome of interest included reported SAE associated with n-3 supplementation. Chi-square analyses were conducted on the pooled aggregate of AEs.

**Results:**

Of the 398 citations initially retrieved, a total of 10 studies involving 994 older adults aged ≥60 years were included in the review. Daily fish oil doses ranged from 0.03 g to 1.86 g EPA and/or DHA with study durations ranging from 6 to 52 weeks. No SAE were reported and there were no significant differences in the total AE rate between groups (n-3 intervention group: 53/540; 9.8%; placebo group: 28/454; 6.2%; *p* = 0.07). Non-SAE relating to gastrointestinal (GI) disturbances were the most commonly reported however there was no significant increase in the proportion of GI disturbances reported in participants randomized to the n-3 intervention (n-3 intervention group: 42/540 (7.8%); placebo group: 24/454 (5.3%); *p* = 0.18).

**Conclusions:**

The potential for AEs appear mild-moderate at worst and are unlikely to be of clinical significance. The use of n-3 fatty acids and the potential for SAE should however be further researched to investigate whether this evidence is consistent at higher doses and in other populations. These results also highlight that well-documented data outlining the potential for SAE following n-3 supplementation are limited nor adequately reported to draw definitive conclusions concerning the safety associated with n-3 supplementation. A more rigorous and systematic approach for monitoring and recording AE data in clinical settings that involve n-3 supplementation is required.

## Background

There is evidence for benefits of long chain omega-3 (n-3) fatty acids in pathological inflammation [1] including rheumatoid arthritis [2], crohn’s disease [3], ulcerative colitis [4,5], diabetes mellitus [6], cardiovascular events [7-9] and cancer cachexia [10]. For this reason, various organizations around the world have established dietary recommendations and guidelines for the intake of n-3 fatty acids (in particular for eicosapentaenoic acid (EPA) and docosahexaenoic acid (DHA)) that are focused primarily on the reduction of cardiovascular disease (CVD) risk [11]. In Australia and New Zealand, the National Health and Medical Research Council (NHMRC) suggest a dietary target for the reduction of chronic disease of 610 and 430 mg/day DHA + EPA for males and females respectively [12,13]. For the prevention of CVD, the National Heart Foundation (NHF) of Australia recommends a dietary intake of 500 mg/day EPA + DHA, the equivalent of two to three servings (150 grams) of oily fish per week [13]. For adults with diagnosed CVD, a combined dosage of 1000 mg EPA + DHA per day is recommended which is best achieved by fish oil supplementation [13].

Fish oil supplementation has become an established complementary medicine (CM). Data taken from the 2004–05 Australian National Health Survey (NHS) indicated that approximately one-quarter (~1.3 million) of Australian adults affected by chronic illness regularly used some form of CM with users more likely to be ≥60 years and female [14]. Cross-sectional analyses from the Australian Longitudinal Study of Ageing (ALSA) showed that cod liver and fish oils were the most frequently used nutritional supplement (excluding vitamin and mineral supplementation) amongst older adults aged ≥65 years [15].

Despite many reviews concentrating on the efficacy of n-3 fatty acids in various conditions [16-20], the potential for serious adverse events (SAE) is not well documented, particularly in older adults. The most commonly raised concern for administration of n-3 fatty acids is their potential to increase the risk of serious bleeds through their anti-platelet effects [21-26]. If there is systematic evidence for serious bleeds or other SAE, the widespread use of n-3 supplementation amongst older adults makes this a public health concern. At the present time, fish oil use as a supplement or CM remains largely unregulated in Australia and currently there are no quantity restrictions of key ingredients or advisory statements concerning these ingredients for labelling purposes [27]. Because many n-3 fatty acid preparations are largely marketed as food supplements, their potential to contribute to SAE is often overlooked.

The aim of this systematic review was to assess published peer reviewed literature of randomized controlled trials (RCTs) to identify the potential for SAE and non-serious adverse events (non-SAE) associated with n-3 supplementation in older adults.

## Methods

### Criteria for considering studies for this review

#### Types of studies

All studies included in this review were RCTs that included the oral supplementation of either liquid fish oil or fish oil capsules, containing EPA, DHA or both, versus a placebo.

#### *Search methods for identification of studies*

##### Electronic searches

We conducted a comprehensive search strategy using the following electronic databases including MEDLINE (1948 to September 2011), EMBASE (1980 to March 2011), CINAHL (1982 to March 2011), SCOPUS (2004 to March 2011) and THE COCHRANE CENTRAL REGISTAR of CLINICAL TRIALS (CENTRAL). A search strategy using free text and MeSH terms was designed for the retrieval of studies. Search terms included fish oil, n-3 fatty acids, n-3, EPA, DHA, polyunsaturated fatty acids (PUFA), long chain fatty acids, geriatrics, elderly, older adults and aged care. The search strategy was limited to age (≥ 60 years), human studies, restricted to the English language and RCTs. All electronic databases were searched commencing in February 2011 ceasing in September 2011.

#### *Eligibility criteria*

The criteria for selecting publications for this review included:

•Elderly persons with a reported mean age ≥ 60 years. Trials that included younger participants were included if the mean age minus one standard deviation was greater than 60 years. Publications that did not report mean age were excluded.

•Ingestion of liquid fish oil or fish oil capsules

•Study design being a RCT

The exclusion criteria for this review included:

•Review articles and individual patient case studies

•Dietary studies including fish intake or functional foods containing EPA and/or DHA

•Enteral or Total Parenteral Nutrition (TPN) administration of EPA and/or DHA

•Participants with known cardiovascular or metabolic disease, malignancy, renal or liver disease at study entry

•Participants with known CVD risk factors (e.g. hypertension, hypertriglyceridemia, hypercholesterolemia and type 2 diabetes mellitus) at study entry.

•Non-English language

#### *Critical appraisal & evaluation of the literature*

##### Selection of studies

An initial screen of titles and abstracts independently by two review authors (AV and MM) rejected studies that clearly did not meet the inclusion/exclusion criteria. Publications which were unclear from the title or abstract remained in consideration. A final round of evaluation was performed by the same two review authors who independently reviewed each article (full text) against the defined inclusion/exclusion criteria. The reviewers resolved any disagreement by consensus. For publications that passed the final round of evaluation, the same two review authors identified the primary outcomes of interest, i.e. SAE and/or non-SAE following the oral administration of n-3 fatty acid supplementation.

#### *Intensity classification of adverse events*

For the purposes of the present review, reported AEs from all reviewed studies were classified as either SAE or non-SAE. The review authors defined SAE as death, stroke, bleeding or major bruising. Non-SAE were identified as gastrointestinal (GI) disturbances (vomiting, halitosis indigestion, pain, flatulence, diarrhoea, nausea or eructation). Identification and documentation of AEs in each of the reviewed articles were self-reported by study participants and/or reviewed by the clinical investigators in each of the reviewed studies.

#### *Data extraction and management*

Using a pre-designed data extraction form, two review authors (AV and MM) evaluated the selected studies for review. The data extraction form was piloted against three publications in the present review and relevant changes were made to the form. The remaining studies were then evaluated. The data extraction form included a description of the types of participants, the dose and duration of EPA and/or DHA supplementation, the method of measuring adherence to the EPA and/or DHA intervention as well as the aim/s and primary findings for each of the reviewed studies. Any differences in the extraction of data where resolved by referring back to the original article followed by discussion and consensus between all review authors.

#### *Correspondence with authors*

An email addressed to the corresponding author from all reviewed studies was sent requesting any documented AEs throughout the duration of the study that were not reported in the full-text article. We used an excel spread sheet highlighting SAE and non-SAE and requested authors to identify which AEs (if any) were reported in their study and the number of participants affected. We also requested that authors identify any additional AEs that were not highlighted by our correspondence.

#### Assessment of methodological quality of the included studies

The Cochrane Collaboration’s tool for assessing risk of bias was used by two review authors (AV and MM) to evaluate all included studies in the present review. This tool assesses the randomization process (sequence generation, allocation concealment and blinding), completeness of the data collection (for primary outcomes only) and the presence of selective reporting. The methodological quality was further assessed using the Jadad scoring scale [28]. This instrument was developed to assess RCTs by ranking them (scale from 0 to 5) against the likelihood of bias in three item areas which include randomization, double blinding and withdrawals/dropouts [28].

#### Statistical analysis

Using a 2 × 2 contingency table, chi-square analysis using Yates’ correction for continuity were conducted on the pooled aggregate of SAE and non-SAE to identify significant differences in the frequency of reported AEs between groups. All data were analysed using GraphPad InStat version 3.10 for Windows (GraphPad software, San Diego California, United States of America).

## Results

### Results of the search

**Figure 1:**
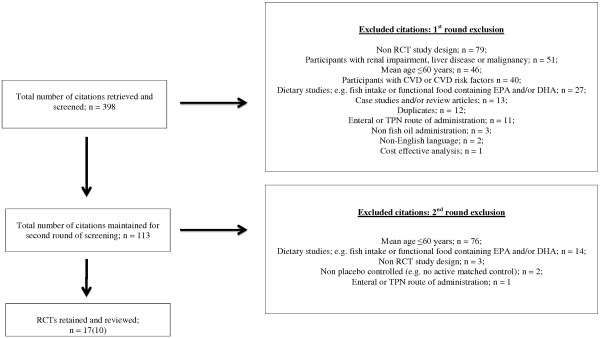
Results of the search strategy for the retrieval of reviewed articles.

Of the 398 citations initially retrieved through the search strategy, a total of 113 articles were maintained for a second round of screening. After reading the full-text articles, the second round of screening removed a further 96 articles. The reasons for rejection in the first and second screening stages are shown in Figure 1.

A total of 17 full-text articles met the inclusion criteria. However, 11 of the 17 full-text articles were duplicate publications: two studies were reported on two occasions in separate articles [29-32], one study was reported on three occasions in separate articles [33-35] and one study was reported on four occasions in separate articles [36-39]. In the present review, results reported from the same trial and population sample and were treated as a single publication source. Therefore, a total of 10 studies were included in the final review (Table 1).

### Trial selection and sample characteristics

The reviewed studies originated from France (n = 2), the United Kingdom (n = 2), the Netherlands (n = 2), Italy (n = 1), Sweden (n = 1), Mexico (n = 1), and Iran (n = 1). All trials were published between 1999 and 2011. The total number of participants in all reviewed studies was 994 (n-3 treatment group n = 540) with a reported mean age of 73.5 years.

### Study durations and n-3 doses

The duration of the studies reviewed ranged from 6 to 52 weeks. Intervention doses ranged from 0.03 g to 1.86 g EPA and/or DHA per day. In 8 of 10 studies n-3 fatty acids were administered via capsules. Rondanelli et al. 2010 [40] administered n-3 fatty acids as a liquid. The method of n-3 fatty acid administration was not specified by Querques et al. 2009 [41].

### Reported AEs from included studies

#### Reported SAE

No SAE attributable to the n-3 intervention were reported. Mortality (2 cases) was reported in one study only (Van de Rest et al. 2009) [36-39] however the treatment allocation was not disclosed. These same investigators also reported a transient ischemic attack (TIA) (1 case) in the placebo arm [36-39]. Gruenwald et al. 2009 [42] reported a total of 21 AEs (21/177; (11.8%) throughout the study duration including 9 that were described as being of “stronger intensity”. The authors did not differentiate AEs by treatment allocation, however it was reported that all AEs were not severe and an association between intake of the investigational product and reported AEs was “unlikely”.

#### *Reported non-SAE*

All non-SAE are shown in Table 2. GI disturbances were the most commonly reported AE with five studies [32,36,40,43,44] reporting GI disturbances (Table 2). Although GI disturbances were generally reported equally between the n-3 intervention and placebo groups, Holguin et al. 2005 [44] reported significantly more cases of eructation in participants randomized to the n-3 intervention group (n-3 intervention: 11/26 (42.3%); control group: 4/26 (15.4%); *p* = 0.04). Freud-Levi et al. 2006 [33-35] reported 9 cases (9/174; 5.2%) of GI disturbances and an additional 9 cases of dysphagia secondary to the size of the study capsules, however the authors did not differentiate the occurrence of GI disturbances between treatment allocations. No studies reported vomiting or halitosis.

#### *Other reported AEs*

Four of 10 studies [36,40,42,43] reported ‘other’ AEs that were not initially considered by the review authors. These included headache, skin irritation, vertigo, malaise, weight gain, polyurination, restlessness, blurred vision, sore throat and skeletal muscle pain. The reported frequencies of these AEs (Table 2) were very low and unlikely to be attributable to the n-3 intervention alone.

Four authors [31,33,36,45] who responded via email correspondence reported no additional AEs than those initially presented in the full-text article.

#### *Anticoagulation exclusions*

Four of 10 studies reviewed excluded participants medicated with oral anticoagulants. Fakhrzadeh et al. 2010 [43] excluded participants taking warfarin ≤30 days prior to study entry. Freund-Levi et al. 2006 [33-35] excluded participants taking any form of oral anticoagulant medication, whilst Holguin et al. 2005 [44] only excluded participants receiving oral anticoagulant medication other than aspirin. Investigators from Thies et al. 2001 [31,32] excluded participants taking any form of prescribed medication, including aspirin.

**Table 1:** Characteristics of included studies

**Author, year**	**Mean age (y)**	**n-3 dosage (EPA/DHA)**	**Control/placebo**	**Duration**	**Sample size ( *n *)**	**Method of administration**
Rondanelli et al. 2010 [40]	84.9	1.67 g/d EPA; 0.83 g/d DHA	Paraffin oil	8 weeks	46	Liquid

^a^ Bechoua et al. 2003 [29]	75.6	30 mg/d EPA 150 mg/d DHA	Sunflower oil	6 weeks	20	Capsules
^b^ Vericel et al. 1999 [30]						

Gruenwald et al. 2009 [42]	62.3	0.6 g/d EPA/DHA	Glucosamine sulfate	26 weeks	177	Capsules

^a^ Van de Rest et al. 2009 [36]	69.8	1.8 g/d EPA/DHA (high-dose); **OR** 0.4 g/d EPA/DHA (low dose)	High-oleic sunflower oil	26 weeks	302	Capsules
^b^ Bouwens et al. 2009 [39]						
^c^ Van de Rest et al. 2008 [37]						
^d^ Van de Rest et al. 2008 [38]						

Querques et al. 2009 [41]	72.7	0.72 g/d EPA; 0.48 g/d DHA	Usual care	26 weeks	38	Unclear

^a^ Thies et al. 2001 [31]	55-75	0.7 g/d DHA; **OR** 0.72 g/d EPA; 0.28 g/d DHA	α-linolenic acid, γ-linolenic acid, **OR** arachidonic acid	12 weeks	46	Capsules
^b^ Thies et al. 2001 [32]						
^a^ Vedin et al. 2010 [33]	74	0.6 g/d EPA; 1.72 g/d DHA	Corn oil	52 weeks	174	Capsules
^b^ Vedin et al. 2008 [34]						
^c^ Freund-Levi et al. 2006 [35]						

Smith et al. 2011 [45]	71	1.86 g/d EPA; 1.5 g/d DHA	Corn oil	8 weeks	15	Capsules
Fakhrzadeh et al. 2010 [43]	74.8	180 mg/d EPA; 120 mg/d DHA		26 weeks	124	Capsules
Holguin et al. 2005 [44]	76.5	2 g/d total marine triglycerides; each 1 g containing 83.2% EPA/DHA	Soy oil	26 weeks	52	Capsules

Studies identified by superscripted numbers refer to those derived from the same trial and population sample and are therefore treated as a single publication source for the purposes of this review.

#### *Overall AE rate*

There was no significant difference (*p* = 0.07) in the total AE rate for participants randomized to the n-3 intervention (53/540; 9.8%) compared with those participants randomized to the placebo arm of studies (28/454; 6.2%). Furthermore, sub-group analyses of pooled non-SAE related to GI disturbances showed no significant increase in the proportion of reported GI disturbances among participants randomized to the n-3 intervention (n-3 intervention: 42/540 (7.8%); placebo group: 24/454 (5.3%); *p* = 0.18).

#### *Compliance to the n-3 intervention*

Seven of 10 studies reviewed showed an increase in measures of n-3 fatty acids (EPA and/or DHA) from either serum, plasma or red blood cell analyses from baseline, post-intervention [29,31,33,36,40,41,45]. Two studies (Gruenwald et al. 2009 [42]; Holguin et al. 2005 [44]) assessed compliance by capsule count. Holguin et al. 2005 [44] reported 93.5% compliance whereas the investigators from Gruenwald et al. 2009 [42] reported only 44% compliance among participants randomized to the n-3 intervention (Table 1).

**Table 2:** Minor reported AEs from reviewed studies

**Minor AEs**	**Authors**	**Fish oil**	**Placebo**
**GI disturbances**	Rondanelli et al. 2010 [40]	6/22 (27.3%)	5/22 (22.7%)
	Van de Rest et al. 2009 [36-39]^a^	High dose: 10/96 (10.4%)	12/106 (11.3%)
		Low dose: 9/100 (9%)	
	Thies et al. 2001 [31,32]	1/8 (12.5%)	-
	Fakhrzadeh et al. 2010 [43]	2/62 (3%)	1/62 (1.5%)
	Holguin et al. 2005 [44]	14/26 (53.8%)	6/26 (23.1%)
**Headache**	Rondanelli et al. 2010 [40]	-	1/24 (4%)
**Skin irritation**	Gruenwald et al. 2009 [42]	2/90 (2%)	-
	Van de Rest et al. 2009 [32-35]^a^	-	1/106 (0.9%)
**Vertigo**	Fakhrzadeh et al. 2010 [43]	1/62 (1.5%)	-
**Malaise**	Fakhrzadeh et al. 2010 [43]	1/62 (1.5%)	-
	Van de Rest et al. 2009 [32-35]^a^	Low dose: 1/100 (1%)	-
**Weight gain**	Van de Rest et al. 2009 [32-35]^a^	High dose: 1/96 (1%)	-
**Polyurination**	Van de Rest et al. 2009 [32-35]^a^	High dose: 1/96 (1%)	-
**Restlessness**	Van de Rest et al. 2009 [32-35]^a^	High dose: 1/96 (1%)	-
**Blurred vision**	Van de Rest et al. 2009 [32-35]^a^	Low dose: 1/100 (1%)	1/106 (0.9%)
**Sore throat**	Van de Rest et al. 2009 [32-35]^a^	Low dose: 1/100 (1%)	-
**Skeletal muscle pain**	Van de Rest et al. 2009 [32-35]^a^	Low dose: 1/100 (1%)	1/106 (0.9%)

^a^ Investigators from Van de Rest et al. 2009 [36-39] included 2 intervention doses of n-3 fish oil; high dose: 1.8 g/d EPA/DHA; low dose: 0.4 g/d EPA/DHA.

#### *Methodological quality of included studies*

Attention toward adequate sequence generation, allocation concealment and complete information for outcome data varied across studies. While the majority of studies provided a clear outline of the progress of participants through the trial (i.e. numbers contributing to analyses at each outcome assessed), the majority of studies lacked detail on why participants withdrew and the relevant characteristics of those participants lost to follow-up. The majority of studies did not clearly identify adequate sequence generation and therefore the trials were largely recorded as ‘unknown’. The most common source of other potential bias was marked differences in baseline characteristics between the intervention and placebo groups**.** The investigators from Van de Rest et al. 2009 [36-39] were the only investigators to adequately address all components of a high quality methodological study design. In contrast, the remaining 9 studies were of a moderate-lower quality in their attention toward the methodological study design (1 study scoring 5/5, 6 scoring 3/5 and 3 scoring 2/5 according to the Jadad system).

## Discussion

We systematically reviewed the potential for AEs in a population of older adults following an intervention of n-3 fatty acid supplementation versus a placebo. Our results are consistent and in support of current literature showing no evidence of SAE associated with oral n-3 supplementation in older adults at doses ≤1.86 g EPA and/or DHA per day. Although non-SAE related to GI disturbances were observed, these were infrequent and not solely apparent in the n-3 intervention group.

Current literature in support of an association between intake of n-3 fatty acids and SAE in an older adult population is scant. In the present review, Van de Rest et al. 2009 [36-39] were the only investigators to report on mortality; however this was deemed unrelated to the study design or n-3 intervention. These same investigators also reported one case of a TIA in the placebo arm of the trial, which was high oleic sunflower oil [36-39]. No studies in the current review reported major bleeds, stroke or bruising. Of clinical importance, 14 of the 17 full-text articles reported positive benefits associated with n-3 supplementation in otherwise healthy older adults; these included reduced symptoms of depression [40], cell-mediated immunity [29,31,46], a reduction in oxidative stress [30], relief from osteoarthritic symptoms [42], anti-inflammatory [33,34,39], anti-atherogenic [39], increased muscle anabolic signalling [45], cardiovascular benefits [43,44] and feasibility and acceptability of n-3 fatty acids [41]. With exception to Holguin et al. 2005 [44] who reported a greater prevalence of GI disturbances (eructation and nausea; ~54%) in participants randomized to the n-3 intervention group, the AE rate was relatively low in all other reviewed studies suggesting that the potential benefits associated with moderate-low dose n-3 supplementation exceed the potential for SAE. Moreover, no studies in the current review reported negative outcomes post-intervention.

The potential for undesirable anticoagulant effects with the concurrent use of fish oil and anticoagulant or antiplatelet medications have previously been highlighted [25,26]. It is speculated that older adults in particular may have an increased risk of major bleeding due to increased sensitivity to anticoagulation, multiple comorbidites and polypharmacy [25]. However, evaluation of the evidence concerning the safety considerations associated with n-3 supplementation shows little support of an increase risk in bleeding with n-3 intake, even when concurrently administered with anticoagulant agents [24]. Despite this Bays 2007 [24] suggested it would be prudent to discontinue high doses of n-3 fatty acid supplementation in the days prior to invasive surgical procedures or in patients at high risk for haemorrhagic stroke. Expert opinion by Harris 2007 [47] was also in agreement suggesting that n-3 fatty acid supplementation does not increase the risk for clinically significant bleeding with the proposed cardiovascular benefits outweighing the theoretical risks for increased bleeding. In the present review only four of ten studies excluded participants at study entry medicated with oral anticoagulant or antiplatelets despite the theoretical potential for anticoagulation of n-3 fatty acids. Regardless of the previous speculation, current evidence has shown that n-3 supplementation at doses ≤4 g per day, even when concurrently administered with antiplatelet or anticoagulant medications, are safe for general consumption [21,48]. However, the doses of n-3 fatty acids in the present review were considerably smaller compared with those previously reported suggesting caution may need to be applied when administering higher doses until further evidence is obtained. Although the majority of studies in the present review (8/10 studies) indicated compliance toward the n-3 intervention, a dose–response relationship between n-3 intake and the potential for AE is unclear given the uncertainty associated with the exact quantity of EPA/DHA ingested in the majority of studies.

Despite the lack of evidence supporting previous recommendations concerning cessation of n-3 supplementation, we recognize that the population group in the present review are non-surgical, older adults free from CVD or CVD risk factors and are therefore unlikely to possess the same theoretical complications associated with bleeding tendency as those with a diagnosis of CVD or with CVD risk factors. Furthermore, it must also be noted that while our findings demonstrated no significant differences in the total AE rate between groups, this was based only on a small number of studies (n = 10) of variable quality. Of further importance was the general lack of consistency in the systematic approach for the recording of all AEs in each of the individual studies. Recognizing this as a potential limitation, the authors of the present review attempted to address this by email correspondence to each corresponding author, however much of the AE data reported in the present review and documented in each of the studies are mainly qualitative and therefore may underestimate nor appropriately describe all potential AEs associated with n-3 supplementation. For example, there are some concerns raised over the potential adverse effect on LDL-cholesterol and the subsequent increased susceptibility to oxidation following n-3 supplementation [49-52]. Additionally, despite certain types of large predatory fish including shark, swordfish and king mackerel containing higher sources of methyl mercury, fish oil preparations supplemented in smaller doses (1-3 g/d) are unlikely to pose SAE related to methyl mercury and/or other contaminants including dioxins and polychlorinated biphenyls (PCBs) [24,50,53]. Moreover, the level of mercury and other environmental toxins present in commercially available fish oil preparations are likely to be negligible given the extensive purification process to remove these [24,53]. However, the potential harm from methyl mercury and/or other environmental contaminants subsequent to long-term exposure at higher doses is less well understood.

Minor intolerances to fish oil are not unusual with symptoms including eructation, nausea, aversion to odour and diarrhoea commonly reported [16,24,54,55]. In keeping with this, our review showed that in an older adult population non-SAE related to GI disturbances were the most commonly reported. Despite this, we observed no significant differences in the frequency of reported GI disturbances between groups, suggesting that the fatty acid composition of fish oil (i.e. EPA and DHA) is unlikely to be the contributing factor for non-SAE. Furthermore, the main placebo(s) used in each of the reviewed studies were typically sunflower or corn oil (n-6 fatty acids) which have both previously demonstrated similar non-SAE as described in the present review [56,57]. Results from a recent palatability study in older adults ≥60 years showed that an association between reported non-SAE and n-3 fatty acid supplementation was unlikely [58]. Using various doses of liquid fish oil (10%, 40% and 100%; containing 5.2 g EPA and 3.6 g DHA at 100% concentrations) Yaxley et al. 2011 [58] reported that older adults were unable to distinguish between varying doses of liquid fish oil and most would not cease consumption if non-SAE such as GI disturbances were experienced.

Fish oils are naturally highly unstable, making them susceptible to oxidation and rancidity [24]. For this reason, many individuals are concerned about taking fish oils as they fear unwanted side effects such as eructation and diarrhoea. Episodes of GI disturbances may be minimized by avoiding aerated drinks at the time of ingestion and by consuming fish oil immediately before meals without further (excessive) fluid consumption [54]. Moreover, tolerance can be improved by refrigerating fish oil supplements (particularly liquid fish oil) once opened [24,54]. Results from the present review suggest that it is feasible that non-SAE relating to taste and smell are associated with personal taste and perception, rather than a physiological rationale. Further improvement in the development and manufacturing of fish oil supplements may improve patient tolerance and perception.

## Conclusions

The potential for AEs related to n-3 supplementation in older adults appear mild-moderate at worst, and not of clinical importance. No reports of SAE including bleeding, stroke or bruising were observed. Non-SAE related to GI disturbances were the most commonly reported however these were infrequent and not significantly different when compared against the placebo group. However this review also highlights that well-documented data describing the potential for SAE following n-3 supplementation are limited nor adequately reported to draw definitive conclusions concerning the safety associated with n-3 supplementation. Although these results suggest that a low-moderate intake of EPA/DHA appear safe and acceptable for use in otherwise healthy older adults, caution should be applied when administering larger doses until further evidence is positioned. Our review further highlights that a more rigorous and systematic approach for monitoring and recording AE data in clinical settings that involve the use n-3 supplementation is required.

## Abbreviations

n-3 fatty acids: Omega-3 fatty acids; RCT: Randomized controlled trial; EPA: Eicosapentaenoic acid; DHA: Docosahexaenoic acid; SAE: Severe adverse events; non-SAE: Non-severe adverse events; GI: Gastrointestinal

## Competing interests

None of the authors have any financial or non-financial competing interest to declare. The authors however would like to acknowledge funding provided for the ongoing ATLANTIC randomized controlled trial supported by the National Health and Medical Research Council (NHMRC), Australia.

## Authors’ contributions

All authors contributed to this work. AMV conducted the literature search, conducted the methodological review of studies, interpreted the findings and prepared the manuscript. MDM interpreted the findings, conducted the methodological review and prepared the manuscript. MC, LGC, MJJ, RJF and LC provided intellectual input in refining the review to its final form. A final acknowledgement to Dorothy Goh, an international student of nutritional sciences completing an independent studies project at Flinders University, Adelaide, South Australia who assisted in the methodological review and the extraction of data from reviewed studies. All authors read and approved the final manuscript.

## Pre-publication history

The pre-publication history for this paper can be accessed here:

http://www.biomedcentral.com/1471-2318/13/41/prepub
